# Supplementary material for: New orphan disease therapies from the proteome of industrial plasma processing waste- a treatment for aceruloplasminemia
Source: Commun Biol. 2024 Jan 30;7:140. doi: 10.1038/s42003-024-05820-7 (PMC10828504; doi:10.1038/s42003-024-05820-7)
Supplement: Supplementary file 2 — Supplementary information [file 42003_2024_5820_MOESM2_ESM.pdf]

Supplementary Figure 1

a

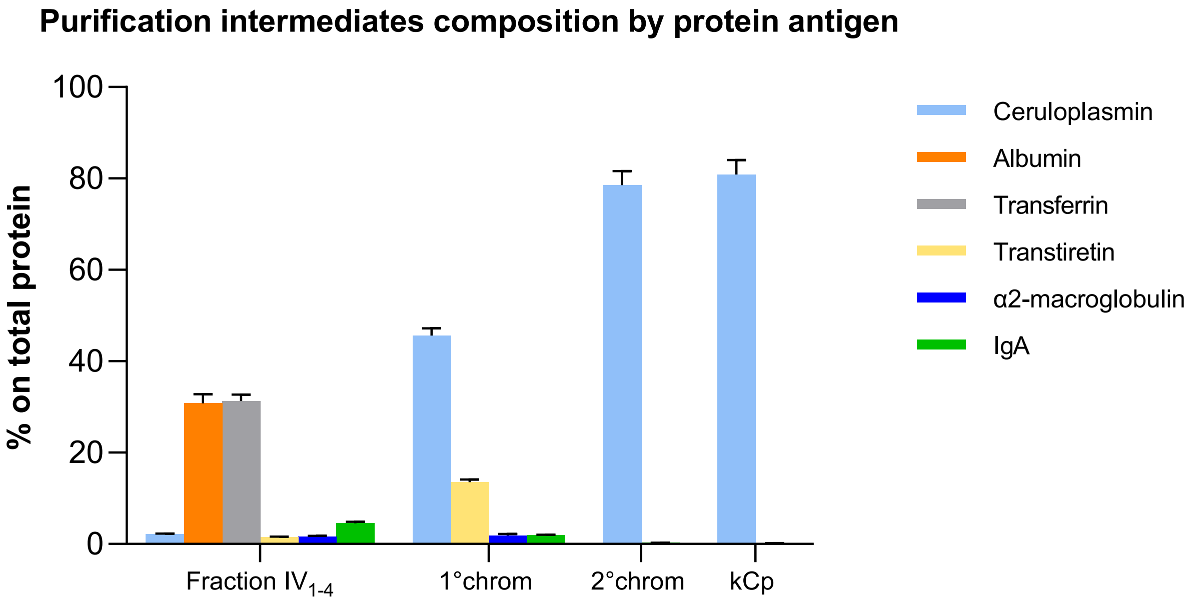

b

INTERMEDIATE	Ceruloplasmin	Albumin	Transferrin	Transthyretin	α2-macroglobulin	IgA
	% on Total protein					
**Fraction IV$_{1-4}$**	**2.11 ± 0.14**	**30.78 ± 1.99**	**31.32 ± 1.41**	**1.50 ± 0.09**	**1.62 ± 0.18**	**4.50 ± 0.38**
**1° Chrom**	**45.59 ± 1.61**	**ND**	**ND**	**1.04 ± 0.52**	**1.88 ± 0.35**	**1.97 ± 0.10**
**2° Chrom**	**78.58 ± 3.01**	**ND**	**ND**	**ND**	**ND**	**0.27 ± 0.03**
**kCP**	**80.85 ± 3.19**	**ND**	**ND**	**ND**	**ND**	**0.14 ± 0.05**

Supplementary Figure 1. Progressive enrichment of CP and removal of the most abundant contaminants (Albumin and Transferrin in the first chromatographic step; Transthyretin, α2-macroglobulin, IgA in the second chromatographic step) during the purification process, starting from FIV$_{1-4}$. (**a**). Summary table indicating relative abundance of each protein in the starting material (FIV$_{1-4}$), intermediates and in the final product (CP concentrate, kCP). Relative levels of each protein antigen with respect to total protein in each sample (± SEM) are shown (**b**).

Supplementary Figure 2

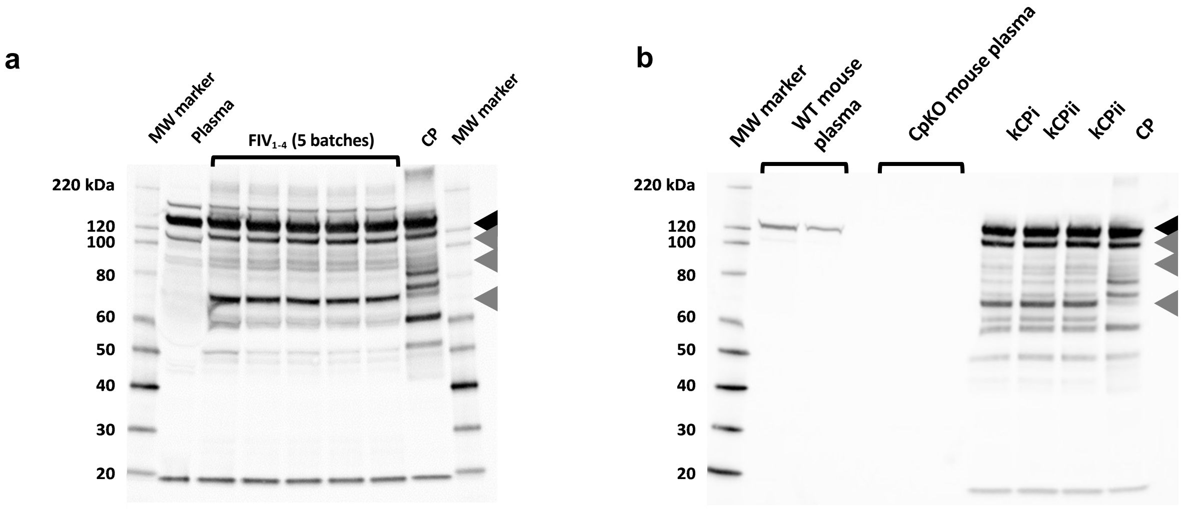

Supplementary Figure 2. Comparability of CP immunoblotting profiles in plasma, FIV$_{1-4}$, kCP and the commercially available CP employed in the published in vivo studies [1,2]. (**a**). Comparability of the CP immunoblotting profile of plasma, FIV$_{1-4}$ (five independently produced batches) and of commercially available CP. A representative blot is shown (N= at least 3). Intact ceruloplasmin has a predicted MW of 132 kDa (black arrowhead). Known protease cleavage sites [3] at R481, R701 and K887 likely result in the production of smaller fragments of 70, 90 and 116 kDa respectively (grey arrows). A representative blot is presented (N= at least 3). (**b**). Specificity of anti-CP polyclonal antibody as determined by analysis of plasma from WT and cpKO mice (two animals for each genotype), and comparison of kCP (2 independent kCP batches kCPi and kCPii, this latter loaded twice) and in commercially available CP.

Supplementary Figure 3

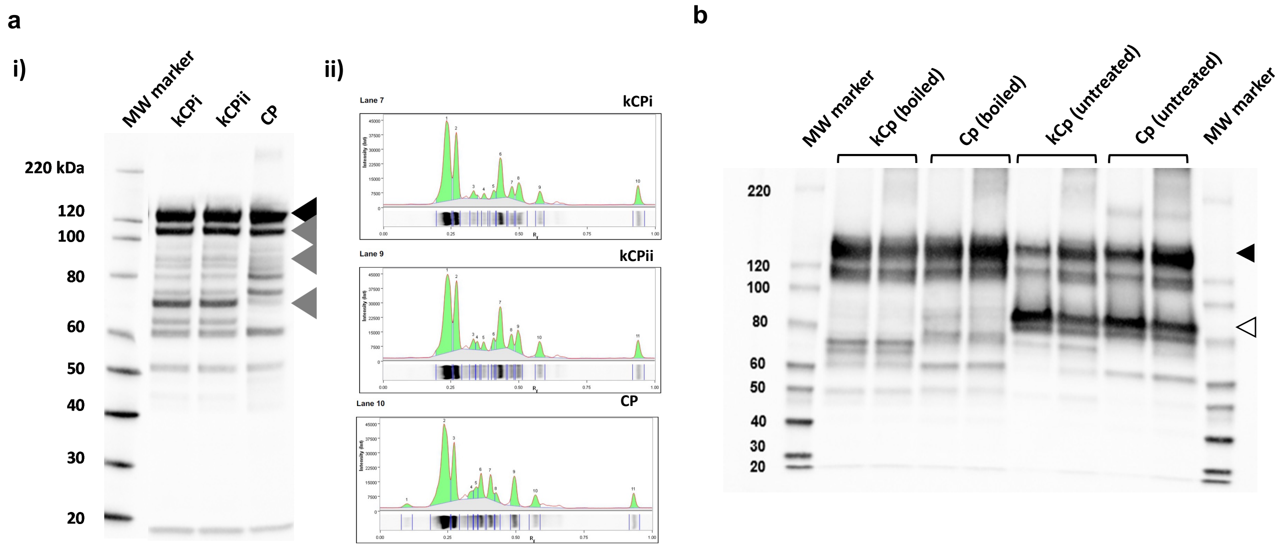

Supplementary Figure 3. Comparison of kCP with the commercially available CP employed in the published in vivo studies [1,2,4]. (**a**). The immunoblotting profiles of kCP (two independently produced batches) and commercial CP are comparable (ai and densitometric comparison in aii). Intact ceruloplasmin has a predicted MW of 132 kDa (black arrowhead). Known protease cleavage sites [3] at R481, R701 and K887 likely result in the production of smaller fragments of 70, 90 and 116 kDa respectively (grey arrows). (**b**). The proportion of apo-CP (solid black arrow) and holo-CP (copper-complexed, white arrow) in kCP and commercial CP are comparable, as demonstrated by a comparison of denatured CP proteins (by boiling prior to loading) vs untreated proteins (e.g. see [4,5]). Representative blots are presented (at least N=3).

Supplementary Figure 4

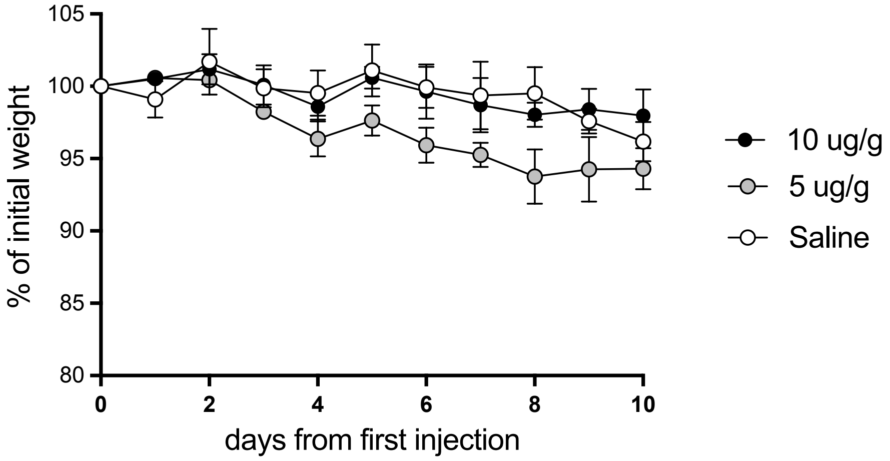

Supplementary Figure 4. Evaluation of acute toxicity induction upon repeated injections of purified kCP. Three groups of 3 cpKO mice of 6 months of age were administered intraperitoneally with saline as control or purified kCP at 5 µg/g, the dosage previously used for the replacement therapy experiments [1], or at double dosage of 10 µg/g. The animals were injected twice, at time 0 and after 5 days and were monitored until day 10 from the first injection. Acute toxicity effects eventually occurring after purified kCP administration were monitored at 1 h, and again 5 h after the injection. Then a daily visual inspection was done for the following days along the entire treatment, and mice body weight was daily recorded. Parameters evaluated during visual inspection were: death, motility, convulsions, apathy to solicitation, crouching, fur alterations (ruffling, loss), and weight. Following the first administration no adverse effects were recorded, nor were any changes in body weight. A similar monitoring schedule was applied after the second administration. At the end of day 10 from the first injection none of the mice showed signs of suffering/distress or anomalous behavior. During the treatment the animals showed body weight variations within the physiological/technical range of +/- 5 % regardless the type of treatment. At the end of the treatment the body weight variations among different groups were not significant (Mann-Whitney test, and Kruskal-Wallis test) and were -3.8 % for the group treated with saline, -5.6 % for mice treated with 5 µg/g kCP and - 2.1% for mice treated with 10 µg/g kCP. Body weight variation in mice injected with saline, or purified kCP, data are presented as means ± SEM. N= 3 for each group.

Supplementary Figure 5

a

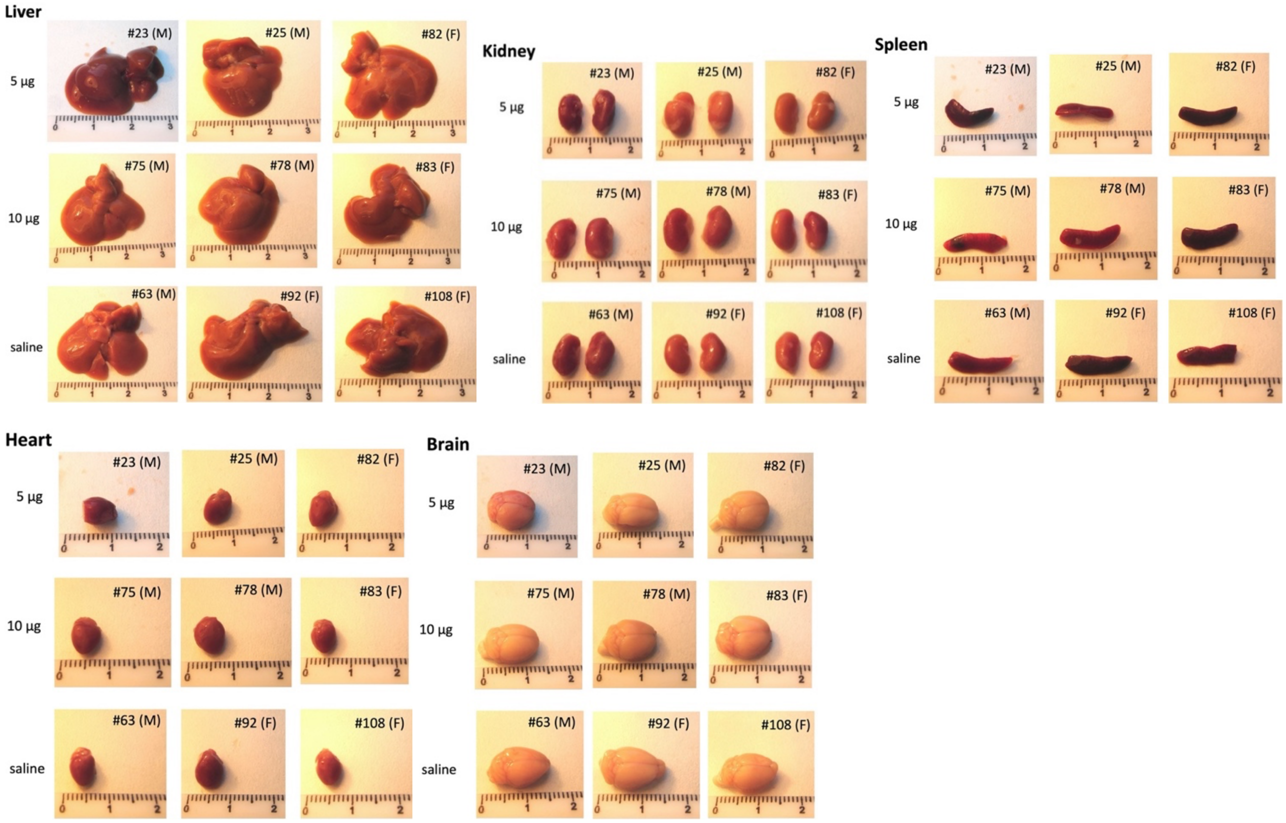

b

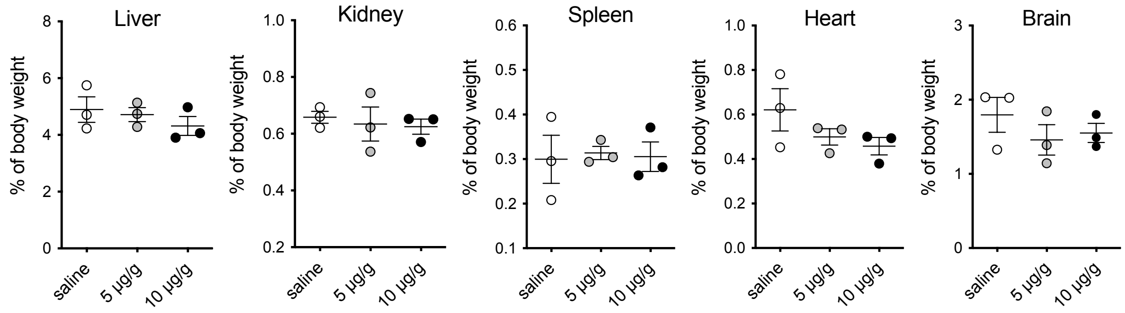

Supplementary Figure 5. Comparison of the organs from mice injected with saline, or purified kCP at 5 and 10 µg/g. Three groups of 3 cpKO mice of 6 months of age were administered intraperitoneally with saline as control or purified kCP at 5 µg/g, the dosage previously used for the replacement therapy experiments [1], or at double dosage of 10 µg/g. The animals were injected twice, at time 0 and after 5 days and were monitored until day 10 from the first injection. At day 10 from the first injection mice were euthanized by perfusion with saline buffer under deep anesthesia and cervical dislocation. Selected organs (brain, liver, heart, spleen, kidney) were collected for macroscopic inspection, weighted and images recorded. **a)** No macroscopic alteration of the organs nor sign of necrosis were detected regardless the type of treatment. **b)** The organs weight, defined as % of the body weight, did not show significant differences among the groups (Mann-Whitney test, and Kruskal-Wallis test). Data are presented as means ± SEM, each dot corresponds to one animal.

Collectively, the results obtained in this pilot study indicated that the kCP purified from $FIV_{1\text{-}4}$ does not exert major toxic effects either in acute or over a short time interval, in cpKO mice when administered at 5 or 10 µg/g every 5 days.

Supplementary Figure 6

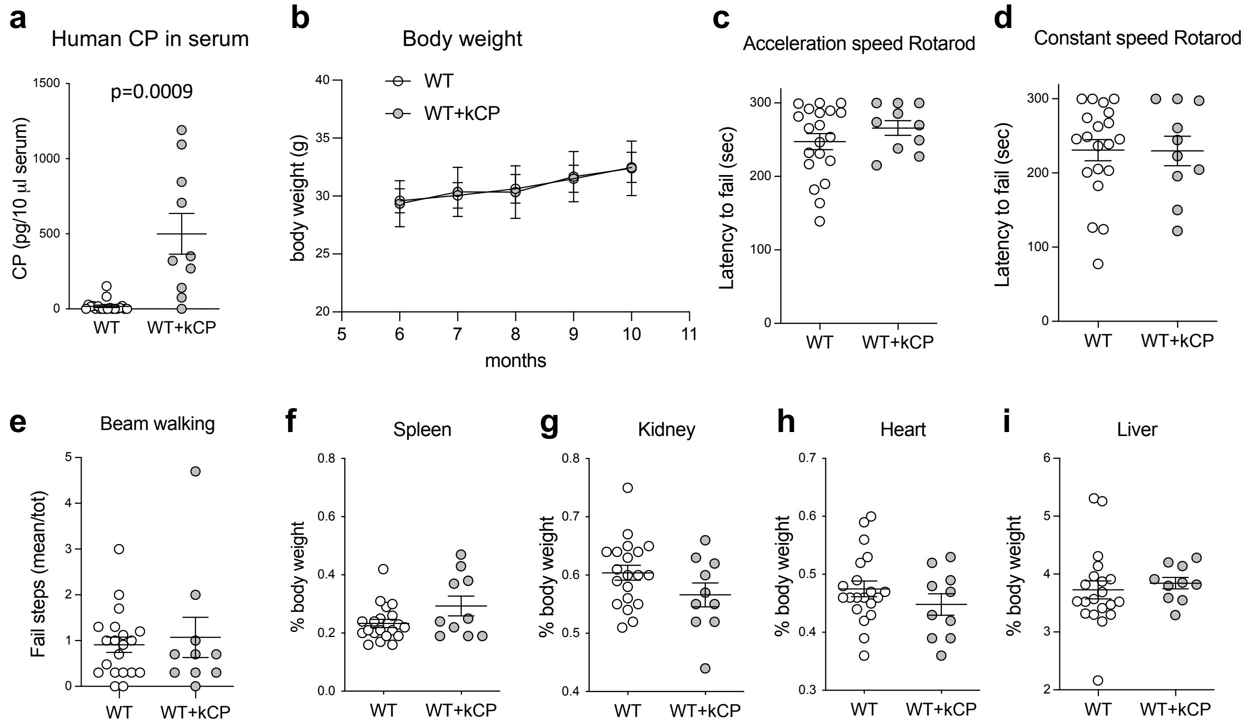

Supplementary Figure 6. Evaluation of toxicity induction in WT mice treated for 4 months with repeated injections of purified kCP. We included in our study a parallel satellite group of WT mice (N=10, 5 males and 5 females), which were treated for 4 months (from 6 to 10 months of age) with purified kCp with the same protocol used for the therapeutic treatment of cpKO mice (5 µg/g kCP administered intraperitoneally every 5 days). In this group of animals we evaluated selected parameters and behaviours, as well as gross signs of toxicity, namely changes in body weight and external appearance (e.g. fur appearance such as smooth/shiny/ruffled/bristly fur), the eyes (clear and shiny, or closed and not clean) and the general state of cleanliness as an index of normal grooming activities. Spontaneous and social behaviour were also observed (normal or reduced activity, isolation from the group) and the response to external stimuli (normal or limited depression or exaggeration of the response). None of these parameters resulted to be altered during the treatment with purified kCp in WT animals as also recorded for cpKO animals of the study. At the end of the treatment WT mice were evaluate for motor coordination behaviour tests (rotarod and beam walking assays) and animals euthanized for major organs (heart, liver, spleen, kidney) collection and evaluation (visual inspection and weight). No significant differences were found in motor coordination and organs appearance/weight of the WT kCp-treated mice in comparison with the parallel group of WT saline-treated animals of the experimental protocol. **a**) ELISA evaluation of human CP levels in the plasma of WT mice treated 4 months with kCP (WT+kCP) compared to the group of WT treated with saline in the experimental study (WT). **b**) Body weight evaluation of the mice from 6 to 10 months of age during the treatment with either purified kCP or saline. Data are reported as means ± SEM of the mice in the two groups. **c-e**) Motor coordination behaviour analysis for (**c**) acceleration speed rotarod, (**d**) constant speed rotarod and (**e**) beam walking test in mice after 4 months of treatment with kCP (WT+kCP) or saline (WT). **f-i**) Organs weight (as % of body weight) of mice at the end of treatment with kCP or saline buffer. Data are presented as means ± SEM, *N*= 10 and N= 20 for WT+kCP and WT group, respectively; each dot corresponds to one animal. Statistical p values were evaluated by Student's t test.

Supplementary Figure 7

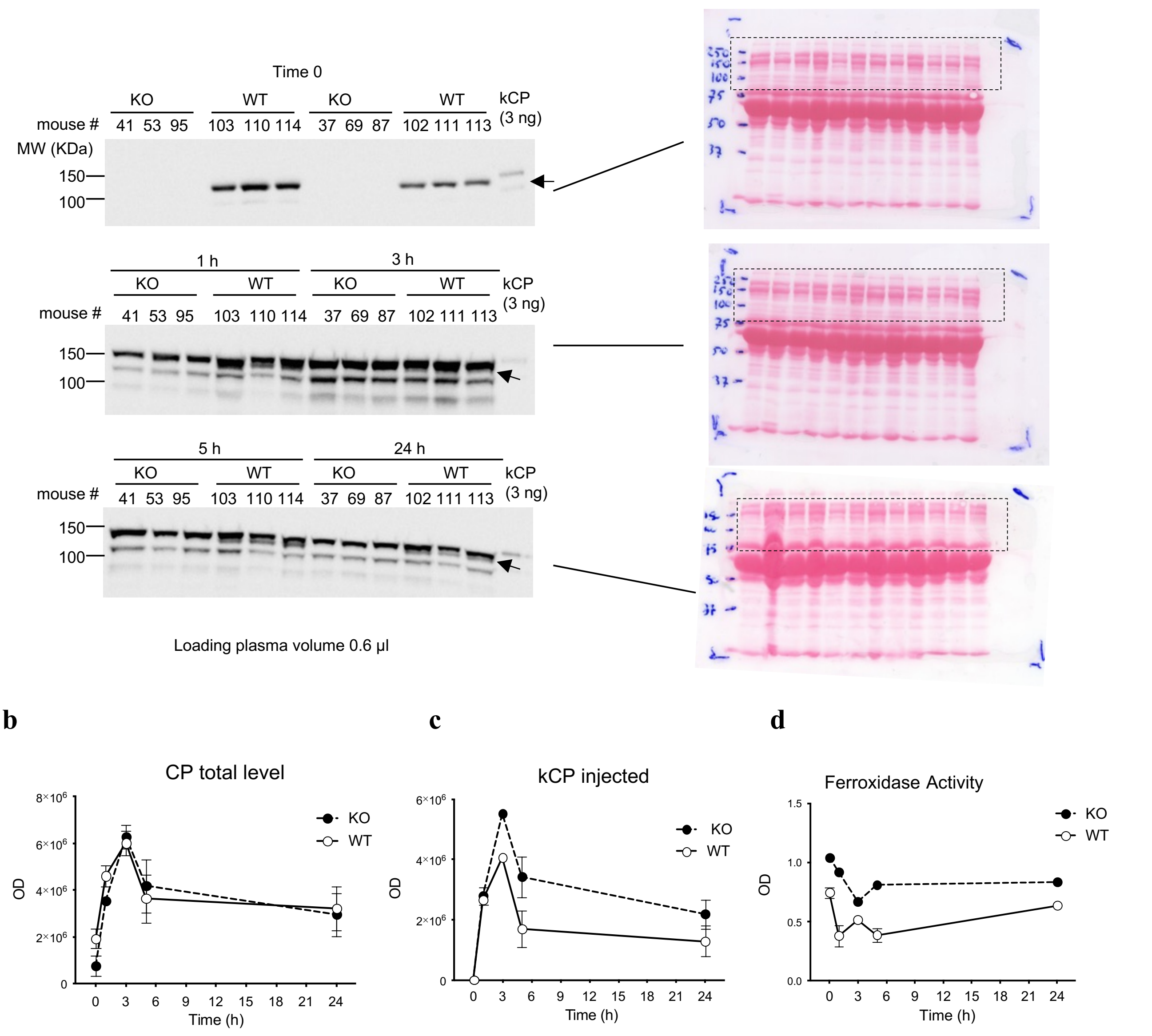

Supplementary Figure 7. Evaluation of the systemic expression of the purified kCP injected in WT and cpKO mice. An evaluation of the systemic administration of kCP was performed on both cpKO mice and C57BL/6J WT mice at 6 months of age (both males and females were used). WT and cpKO mice (n= 6/group) were treated by intraperitoneal injection (5 µg/g) of kCP. Blood (0.1 ml) was collected one week before kCP injection (time 0) and at 4 different time points (1, 3, 5 and 24 h) post-injection. To pace blood draws, collections from 3 mice for each group occurred at 1h and 5h from the kCP injection and the collections from the other 3 mice of the group occurred at 3h and 24h from kCP administration. The kCP level in serum was evaluated by Western blot and densitometric analysis as averages of two technical gel replicates, and the kCP ferroxidase activity was evaluated using the Erel method. **a**) Representative images of the Western blot reactivity for kCP presence in serum of cpKO and WT mice at different time after administration. At time zero only endogenous CP in WT mice was detectable. Purified kCP give rise to two major bands (as also visible in the signals of the standard purified kCP loaded on the right of the gel) that are distinguishable from the endogenous CP signal that is in between of these bands (visible in WT mice, arrows). Panels on the left are the portion of the nitrocellulose membranes that were cut (see dashed rectangle on the right

panels) for probing with anti CP-antibody. **b**) Quantitative evaluation by optical density (OD) analysis of the Western blot reactivity for total CP (injected, plus the endogenous in WT) level normalized for both total protein and standard kCP (3 ng) signal within the gels. Total CP level detection in serum demonstrated similar kinetics in both cpKO and WT mice, with a peak of CP accumulation in the blood at 3 h followed by a rapid decrease at 5 h and residual amount of CP, still detectable, at 24 h from the injection. At time 0 an endogenous CP signal is detectable in WT mice, then after 1 h from the injection CP appears also in cpKO mice and increases in WT mice which show an even greater signal, likely due to the presence of the endogenous CP. **c**) Analysis of the signal of purified kCP. At 3, 5 and 24 h from the injection, considering exclusively the signal of the purified human kCP injected, cpKO mice showed more intense signals, suggesting that in WT mice the kinetics of removal of the administered protein is faster than in cpKO mice. **d**) Analysis of ferroxidase activity. The ferroxidase activity was measured by Erel assay in duplicate experiments in the serum of the mice paralleled the kinetics of the kCP appearance indicating a rescue of the ferroxidase activity in the blood of cpKO mice. Note that in the graphical representation, high OD values correspond to low ferroxidase activity. In spite of similar total CP signal in the serum, ferroxidase activity in cpKO mice was lower than the one detected in WT mice, suggesting that the activity of endogenous CP in WT mice is more efficient. Data are presented as means ± SEM, N= 6 for each group.

Supplementary Figure 8

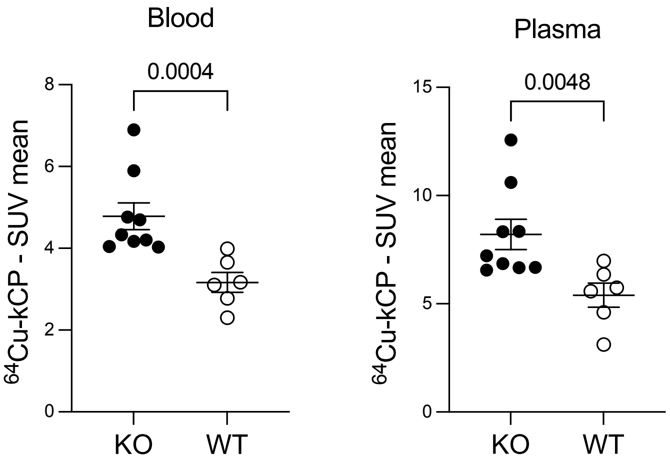

Supplementary Figure 8. *Ex-vivo* analysis of ^{64}Cu-labelled kCP accumulation in blood and plasma of cpKO and WT mice. For CP radiolabelling with ^{64}Cu and protocols for tracer administration and *ex vivo* evaluation se Methods section of the main text. The results indicated that, 21 h post injection in 10 months old cpKO (N=9) and control (N=6) male mice, a significantly higher accumulation of radiolabelled kCP occurred in the blood and plasma of the cpKO mice compared to WT mice ($p=0.0004$, $p=0.0048$, respectively). This observation is in accord with the results of pharmacokinetics in which the amount of injected kCP detectable after 24 h was lower in WT than cpKO mice (see Supplementary Figure 7c). Data are expressed as Standardized Uptake Value (SUV) and are presented as mean ± SEM. Statistical P values were evaluated by Mann-Whitney test and considered significant when <0.05.

Supplementary Table 1

Parameters measured and used in the principal component analysis in addition to those presented in the main text for 6-months old mice.

Parameters	KO	WT	p value
RBC (M/uL)	10.48 ± 0.10	10.49 ± 0.07	0.5993 †
HGB (g/dL)	12.1 ± 0.11	14.8 ± 0.08	< 0.0001
HCT (%)	41.8 ± 0.39	50.8 ± 10.39	< 0.0001 †
MCHC (g/dL)	29.0 ± 0.08	29.2 ± 0.15	0.1926
RDW-SD (fL)	35.6 ± 0.36	30.8 ± 0.35	< 0.0001
RET (%)	3.60 ± 0.13	2.89 ± 0.13	0.0013
PLT (K/uL)	1362 ± 42.09	1032 ± 25.77	< 0.0001

† Mann-Whitney test. RBC: red blood cells; HGB: hemoglobin; HCT: hematocrit; MCHC: mean corpuscular hemoglobin concentration; RDW-SD: red cell distribution width - standard deviation; RET %: percentage of reticulocytes; PLT: platelets. Data are presented as means ± SEM. Statistical p values were evaluated by Student's t-test or by Mann-Whitney test. KO: *N*= 40 (20 male, 20 female). WT: *N*= 20 (10 male, 10 female).

Supplementary Table 2

Hematological parameters measured and used in the principal component analysis in addition to those presented in the main text for 10-months old mice

Parameter	p value	KO	KO + CP	WT
RBC (M/μL)	0.0052	10.70 ± 0.19 [a]	9.98 ± 0.16 [b]	10.56 ± 0.12
HGB (g/dL)	< 0.0001†	12.4 ± 0.26 [b]	12.6 ± 0.17 [b]	15.1 ± 0.13
HCT (%)	< 0.0001†	41.9 ± 0.99 [b]	42.0 ± 0.63 [b]	50.9 ± 0.50
MCHC (g/dL)	0.0167†	29.7 ± 0.12 [a]	30.1 ± 0.12	29.7 ± 0.10
RDW-SD (fL)	< 0.0001	33.5 ± 0.64 [b]	33.3 ± 0.38 [b]	30.0 ± 0.32
RET (%)	0.1504	3.91 ± 0.25	4.44 ± 0.18	4.05 ± 0.15
PLT (K/μL)	0.0007	1507 ± 78.05 [b]	1363 ± 74.82 [b]	1130 ± 36.48
WBC (K/μL)	0.2259†	2.05 ± 0.28	1.58 ± 0.22	1.53 ± 0.28
Cu liver (μg/g dry weight)	0.7940†	24.3 ± 2.82	23.3 ± 1.23	26.8 ± 2.82
Zn liver (μg/g dry weight)	0.3770†	134.1 ± 13.08	139.1 ± 8.07	147.7 ± 12.56
F4/80 staining (a.u.)	0.0106	9.34 ± 0.58 [a,b]	7.27 ± 0.55	7.14 ± 0.55
Spleen (mg)	0.2516	67 ± 2.95	68 ± 2.69	74 ± 2.87
Kidney (mg)	0.6841	192.9 ± 8.95	204.3 ± 10.23	196.3 ± 9.05
Heart (mg)	0.4845	163 ± 6.82	162 ± 7.16	153 ± 5.83
Liver (mg)	0.2581	1313 ± 63.13	1196 ± 64.57	1191 ± 46.25
Cu brain (μg/g dry weight)	0.0221†	19.4 ± 1.64 [b]	22.6 ± 2.42	28.6 ± 3.21
Zn brain (μg/g dry weight)	0.4162†	64.9 ± 6.37	75.7 ± 9.44	82.1 ± 11.54
Triglycerides serum (mM)	0.0628	0.950 ± 0.05	0.820 ± 0.04	0.833 ± 0.04
Adipose tissue weigth (mg)	0.0250	2389 ± 267.2 [b]	1769 ± 147.0	1685 ± 140.5

† Kruskal-Wallis test. [a] = post-hoc test significance *vs.* KO+kCP. [b] = post-hoc test significance *vs.* WT. RBC: red blood cells; HGB: hemoglobin; HCT: hematocrit; MCHC: mean corpuscular hemoglobin concentration; RDW-SD: red cell distribution width - standard deviation; RET %: percentage of reticulocytes; PLT: platelets; WBC: white blood cells. Data are presented as means ± SEM. Statistical p values were evaluated by one-way ANOVA or Kruskal-Wallis test; post-hoc tests used were Tukey's or Dunn's respectively. KO: *N*= 20 (10 male, 10 female). KO+kCP: *N*= 20 (10 male, 10 female). WT: *N*= 20 (10 male, 10 female).

Supplementary References

1) Zanardi, A. et al. Ceruloplasmin replacement therapy ameliorates neurological symptoms in a preclinical model of aceruloplasminemia. *EMBO Mol. Med.* **10**, 91-106 (2018).
2) Ayton, S. et al. Ceruloplasmin dysfunction and therapeutic potential for Parkinson disease. *Ann. Neurol.* **73**, 554-9 (2013).
3) Vasilyev, V.B. Looking for a partner: ceruloplasmin in protein-protein interactions. *Biometals* **32**, 195-210 (2019).
4) Hellman, N.E. and Gitlin J.D. Ceruloplasmin metabolism and function. *Annu. Rev. Nutr.* **22**, 439-58 (2002).
5) Linder, M.C. Apoceruloplasmin: Abundance, Detection, Formation, and Metabolism. *Biomedicines*. **9**(3) (2021).
