# Supplementary material for: New orphan disease therapies from the proteome of industrial plasma processing waste- a treatment for aceruloplasminemia
Source: Commun Biol. 2024 Jan 30;7:140. doi: 10.1038/s42003-024-05820-7 (PMC10828504; doi:10.1038/s42003-024-05820-7)
Supplement: Supplementary file 3 — Description of Additional Supplementary Files [file 42003_2024_5820_MOESM3_ESM.pdf]

Description of Additional Supplementary Files

File name: Supplementary Data 1
Description: Proteomics analysis of plasma processing waste fractions.

File name: Supplementary Data 2
Description: Protein prioritization analysis.

File name: Supplementary Data 3
Description: Prioritized proteins and ceruloplasmin characterization.

File name: Supplementary Data 4
Description: Ceruloplasmin purification.

File name: Supplementary Data 5
Description: Raw data used for PCA analysis.
