# Supplementary material for: New orphan disease therapies from the proteome of industrial plasma processing waste- a treatment for aceruloplasminemia
Source: Commun Biol. 2024 Jan 30;7:140. doi: 10.1038/s42003-024-05820-7 (PMC10828504; doi:10.1038/s42003-024-05820-7)
Supplement: Supplementary file 9 — Reporting Summary [file 42003_2024_5820_MOESM9_ESM.pdf]

nature portfolio

Corresponding author(s): Massimo Alessio, Andrea Caricasole

Last updated by author(s): Dec 15, 2023

Reporting Summary

Nature Portfolio wishes to improve the reproducibility of the work that we publish. This form provides structure for consistency and transparency in reporting. For further information on Nature Portfolio policies, see our Editorial Policies and the Editorial Policy Checklist.

Statistics

For all statistical analyses, confirm that the following items are present in the figure legend, table legend, main text, or Methods section.

n/a	Confirmed	
☐	☒	The exact sample size (*n*) for each experimental group/condition, given as a discrete number and unit of measurement
☐	☒	A statement on whether measurements were taken from distinct samples or whether the same sample was measured repeatedly
☐	☒	The statistical test(s) used AND whether they are one- or two-sided<br>*Only common tests should be described solely by name; describe more complex techniques in the Methods section.*
☒	☐	A description of all covariates tested
☐	☒	A description of any assumptions or corrections, such as tests of normality and adjustment for multiple comparisons
☐	☒	A full description of the statistical parameters including central tendency (e.g. means) or other basic estimates (e.g. regression coefficient) AND variation (e.g. standard deviation) or associated estimates of uncertainty (e.g. confidence intervals)
☐	☒	For null hypothesis testing, the test statistic (e.g. *F*, *t*, *r*) with confidence intervals, effect sizes, degrees of freedom and *P* value noted<br>*Give P values as exact values whenever suitable.*
☒	☐	For Bayesian analysis, information on the choice of priors and Markov chain Monte Carlo settings
☒	☐	For hierarchical and complex designs, identification of the appropriate level for tests and full reporting of outcomes
☐	☒	Estimates of effect sizes (e.g. Cohen's *d*, Pearson's *r*), indicating how they were calculated

Our web collection on statistics for biologists contains articles on many of the points above.

Software and code

Policy information about availability of computer code

Data collection	Proteome Discover 2.5 (ThermoFisher Scientific); Biopharma Finder 2.0 software (Thermo Fisher Scientific);
Data analysis	Prism V9.5.0 software, GraphPad Inc.; ImageJ V2.0.0-rc-43/1-50e; MetaboAnalyst 5.0 online package (www.metaboanalyst.ca/home.xhtml).

For manuscripts utilizing custom algorithms or software that are central to the research but not yet described in published literature, software must be made available to editors and reviewers. We strongly encourage code deposition in a community repository (e.g. GitHub). See the Nature Portfolio guidelines for submitting code & software for further information.

Data

Policy information about availability of data

All manuscripts must include a data availability statement. This statement should provide the following information, where applicable:

- Accession codes, unique identifiers, or web links for publicly available datasets
- A description of any restrictions on data availability
- For clinical datasets or third party data, please ensure that the statement adheres to our policy

"San Raffaele Open Research Data Repository" (ORDR) (https://ordr.hsr.it/research-data/); DOI: 10.17632/r2p5n38mdz.1; the mass spectrometry proteomics data have been deposited to the ProteomeXchangeConsortium via the PRIDE partner repository with the dataset identifier PXD046234.

Human research participants

Policy information about studies involving human research participants and Sex and Gender in Research.

Reporting on sex and gender	The material use in our study are the waste fractions obtained from the industrial processing of plasma batches that are random pools of donations collected as per applicable national and international legislation and industry standards (see Weinstein M Ann Blood 3:3, 2018) which comply the regulations and standards of the United States Food and Drug Administration (FDA) and the voluntary standards of the Plasma Protein Therapeutic Association (PPTA). The batches included plasma from both males and females donors and from multi-ethnic origin.
Population characteristics	The batches included plasma from both males and females donors and from multi-ethnic origin.
Recruitment	NA
Ethics oversight	NA

Note that full information on the approval of the study protocol must also be provided in the manuscript.

Field-specific reporting

Please select the one below that is the best fit for your research. If you are not sure, read the appropriate sections before making your selection.

☒ Life sciences ☐ Behavioural & social sciences ☐ Ecological, evolutionary & environmental sciences

For a reference copy of the document with all sections, see nature.com/documents/nr-reporting-summary-flat.pdf

Life sciences study design

All studies must disclose on these points even when the disclosure is negative.

Sample size	The sample size was established using the G-Power v3.1.9.4 software (Heinrich-Heine-Universität Düsseldorf), applying a one-way ANOVA test for the comparison of means between 3 groups with alpha error of 0.05 and power of 0.8; effect size (Cohen's effect size) of 0.45.
Data exclusions	No acquired data where excluded from the analysis.
Replication	Whenever possible 3 -5 technical replicates for data acquisition were measured for each of the 20 mice/group used as biological replicates.
Randomization	Mice were randomly distributed in the treatment and control group matching sex distribution among groups.
Blinding	Data acquisition and analysis were performed in blind.

Reporting for specific materials, systems and methods

We require information from authors about some types of materials, experimental systems and methods used in many studies. Here, indicate whether each material, system or method listed is relevant to your study. If you are not sure if a list item applies to your research, read the appropriate section before selecting a response.

Materials & experimental systems

n/a	Involved in the study	
☐	☒	Antibodies
☒	☐	Eukaryotic cell lines
☒	☐	Palaeontology and archaeology
☐	☒	Animals and other organisms
☒	☐	Clinical data
☒	☐	Dual use research of concern

Methods

n/a	Involved in the study	
☒	☐	ChIP-seq
☒	☐	Flow cytometry
☒	☐	MRI-based neuroimaging

Antibodies

Antibodies used	Anti-Ceruloplasmin antibody: Abcam, ab19171, lot. GR13392-2; Abcam, ab8813, lot. 859941; Abcam, ab48614; SantaCruz Biotechnology, sc21242, lot. F0903; SantaCruz Biotechnology, sc21240, lot. D0406.<br>Anti-NeuN antibody: Merck Millipore, MAB377.

	Anti-IBA1 antibody: WAKO Chemicals USA, 016-20,001.<br>Anti-GFAP antibody Alexa Fluor 488: Merck Millipore, MAB3402X.<br>Secondary Ab<br>Rabbit anti-goat Ig-HRP: Dako, Agilent P044901-2, lot. 00067644<br>Alexa Fluor 635 anti-rabbit Ig antibody: Thermo Fisher Scientific, A31577.<br>Alexa Fluor 555 anti-mouse Ig antibody: Thermo Fisher Scientific, A31570.
Validation	In addition to the specificity statement and references reported on the website of the commercial antibodies used, all anti-ceruloplasmin antibodies were further validated by Western blot performed on the wild-type and ceruloplasmin-deficent mice sera.

Animals and other research organisms

Policy information about studies involving animals; ARRIVE guidelines recommended for reporting animal research, and Sex and Gender in Research

Laboratory animals	Mus musculus, C57Bl/6J either Wild type or ceruloplasmin knock-out from 6 to 10 months of age were used.
Wild animals	The study did not involve wild animals
Reporting on sex	Since no sex difference in aceruloplasmineamia penetrance or features have been reported in both human and preclinical models, mice of both sex matched in the different groups were used in the study and analysed together.
Field-collected samples	The study did not involve samples collected from the field.
Ethics oversight	The study was approved by the Institutional Animal Care and Use Committee and by the National Ministry of Health (n°77/2020-PR and n°7/2022-PR).

Note that full information on the approval of the study protocol must also be provided in the manuscript.
